# Supplementary material for: MetaRibo-Seq measures translation in microbiomes
Source: Nat Commun. 2020 Jun 29;11:3268. doi: 10.1038/s41467-020-17081-z (PMC7324362; doi:10.1038/s41467-020-17081-z)
Supplement: Supplementary file 10 — Supplementary Data 7 [file 41467_2020_17081_MOESM10_ESM.zip › File2/Confidence_High_Taxonomy/60041_out.krona.html]

Javascript must be enabled to view this page.

members
magnitude
magnitudeUnassigned
count
unassigned
taxon
rank

60041\_out

32

2
32
5
superkingdom

SRS018984\_contig\_number\_contig-80\_1325.246191SRS047044\_contig\_number\_contig-100\_5521.106845SRS052027\_contig\_number\_contig-100\_2569.124363SRS064276\_contig\_number\_contig-100\_3800.220644SRS148319\_contig\_number\_contig-100\_4324.105139

phylum
1

SRS019910\_contig\_number\_12441
27
1239

species

SRS048870\_contig\_number\_contig-100\_9271.86971
1
1897036

186801
class
25


SRS054905\_contig\_number\_contig-100\_4874.151326
order
1
25
186802

14
family
186806

14
genus
1730

4

SRS017433\_contig\_number\_contig-100\_9461.78817SRS049896\_contig\_number\_contig-100\_558.204588SRS147377\_contig\_number\_contig-100\_3461.83482SRS148721\_contig\_number\_34890
species
1262892

39485
8

SRS014313\_contig\_number\_contig-100\_3363.55944SRS014459\_contig\_number\_contig-100\_3414.239456SRS016056\_contig\_number\_contig-100\_16479.16479SRS016541\_contig\_number\_contig-100\_23202.23202SRS047014\_contig\_number\_contig-100\_22914.203114SRS1041037\_contig\_number\_contig-100\_1554.95643SRS1055049\_contig\_number\_contig-100\_2549.99717SRS142890\_contig\_number\_contig-100\_5123.248964
species

142586
species

SRS043667\_contig\_number\_contig-100\_629.46194
1

1

SRS049995\_contig\_number\_contig-100\_3565.225031
species
1897026

1
family
31979

1
genus
1485

2293006
1

SRS023526\_contig\_number\_13835
species

9
186803

SRS014736\_contig\_number\_contig-100\_2642.71258
1
family


SRS144183\_contig\_number\_contig-100\_6839.225800
species
1
39491

1898203
species

SRS077127\_contig\_number\_contig-100\_2621.121489
1

28050
genus
2

28052
species

SRS144135\_contig\_number\_14585SRS146812\_contig\_number\_58122
2


SRS050998\_contig\_number\_contig-100\_3973.112054
genus
1
4
841

species

SRS076756\_contig\_number\_contig-100\_1288.141711
1
2049040

species

SRS046502\_contig\_number\_contig-100\_5286.87916
1
166486

1262947
1
species

SRS104912\_contig\_number\_15647
